# How aware are swingers about their swing sex partners’ risk behaviours, and sexually transmitted infection status?

**DOI:** 10.1186/s12879-021-05813-5

**Published:** 2021-02-12

**Authors:** Anne-Marie Niekamp, Laura W. L. Spauwen, Nicole H. T. M. Dukers-Muijrers, Christian J. P. A. Hoebe

**Affiliations:** 1 Department of Sexual Health, Infectious Diseases and Environmental Health, South Limburg Public Health Service, PO Box 33, 6400 AA Heerlen, the Netherlands; 2 Department of Social Medicine, Care and Public Health Research Institute (CAPHRI), Maastricht University, PO Box 616, 6200 MD Maastricht, the Netherlands; 3 Department of Medical Microbiology, Care and Public Health Research Institute (CAPHRI), Maastricht University Medical Center (MUMC+), PO Box 5800, 6202 AZ Maastricht, the Netherlands

**Keywords:** Swingers, Swing sex partners, Sexually transmitted infections, Bisexual behaviour, Number of sex partners, Network data, Partner data, Sexual health

## Abstract

**Background:**

Swingers are members of a heterosexual couple who, as a couple, have sex with others. They constitute a hidden subpopulation that is at risk for sexually transmitted infections (STIs). This study aimed to determine swingers’ level of awareness about the STI risk (indicators: bisexual behaviour, number of sex partners, and STI status) of their swing sex partners (i.e. *alters*).

**Methods:**

In this cross-sectional study, data were collected from a convenience sample of swingers who visited our STI clinic. The sample consisted of 70 participants (i.e. *egos*) and their 299 swing sex partners (i.e. alters) who had undergone an STI test at our clinic. We compared network data (i.e. information that egos provided about alters) and data stored in the electronic patient record (EPR) in our clinic (i.e. information provided by alters themselves). We assessed the agreement (correct estimation, overestimation and underestimation) between the network data and EPR data using chi-squared tests.

**Results:**

Egos underestimated the bisexual behaviours of 37% of their male alters and overestimated the number of sex partners of 54 and 68% of their male and female alters, respectively. Egos correctly estimated the STI statuses of only 22% of the alters who had an STI during the past six months.

**Conclusions:**

The participating swingers underestimated the bisexual behaviours of their male swing sex partners, overestimated their number of sex partners, and underestimated their positive STI status. Underestimating their alters’ STI statuses can cause swingers to underestimate their own STI risk and fail to implement preventive measures. The latter finding has implications for STI prevention. Therefore, more attention should be paid to swingers in general and the promotion of actual partner notification and STI testing among swingers in specific.

**Supplementary Information:**

The online version contains supplementary material available at 10.1186/s12879-021-05813-5.

## Background

The transmission of sexually transmitted infections (STIs) is influenced by the sexual behaviours of individuals and their sex partners as well as their number of sex partners [1]. Furthermore, their perceptions of their sex partners’ risk behaviours (perceived or estimated risk) influence their implementation of preventive measures [2–4]. For STI prevention to be effective, estimated risk should correspond to actual risk. Therefore, researchers in the field of STI transmission and care are interested in ascertaining the extent to which individuals possess accurate information about the sexual behaviours of their sex partners (i.e. both regular and casual). However, it is challenging to collect data about sexual behaviours because this data is vulnerable to biases (e.g. recall and social desirability bias) [5, 6]. Collecting such data about one’s sex partners is even more challenging.

Several studies have evaluated the accuracy of the information that individuals provide about the sexual behaviours or STI statuses of their sex partners [2, 3, 7–20]. These studies examined the level of agreement between two types of behaviours. Some studies assessed behaviours and risk factors that were shared by a couple (e.g. frequency of having sex together, condom use) and reported good agreement between partners [3, 9, 10, 16–20]. Other studies assessed behaviours and risk factors that a couple did not necessarily share (e.g. concurrency of sex partners, HIV status) [2, 3, 7–13, 15, 20]. While most of the latter studies examined heterosexual couples in committed relationships, they reported low agreement between partners. To the best of our knowledge, no such study has been conducted among swingers.

Swingers are members of heterosexual couples in committed relationships who, as a couple, have sex with other couples and/or singles. They constitute a hidden sexual subpopulation that is at risk for STIs [21–28]. Our study aimed to determine the degree to which swingers are aware of their swing sex partners’ STI risk. We identified indicators of STI risk that are relevant to the swinger population. Couples may not share these risk factors or engage in such risk behaviours together. They were as follows: bisexual behaviour, number of sex partners, and STI status. Although swingers are members of heterosexual couples and identify as heterosexual, many of them engage in same-sex sexual behaviours; therefore, they are bisexual by behaviour [25, 28]. In particular, same-sex sexual behaviours between men are related to a high prevalence of STIs. Further, as implied by the definition, swingers have multiple sexual partners [28]. In a past study, we found that partner notification was the norm among a majority of the participating swingers [28]. Therefore, we hypothesised that most swingers would be aware of each other’s STI statuses.

To address the aim of this study, we examined dyads that consisted of a swinger and his/her casual swing sex partner (i.e. not the partner with whom he/she shared a committed relationship). In this article, we refer to swing sex partners as *alters* to differentiate them from sex partners in general. The participating swingers are referred to as *egos*. We examined the level of agreement between ego-estimated and self-reported STI risk among alters. The following risk factors were assessed: bisexual behaviour, number of sex partners, and STI status.

## Methods

### Study population

The cross-sectional data used in this study were collected as a part of a prospective cohort network study of swingers (i.e. the SWAP study) [21]. A convenience sample of swingers who visited our STI clinic in the south of the Netherlands between 2009 and 2012 constituted the group of egos. Clinic attendees who were self-reported swingers as per the aforementioned definition were eligible for inclusion.

**Fig. 1:**
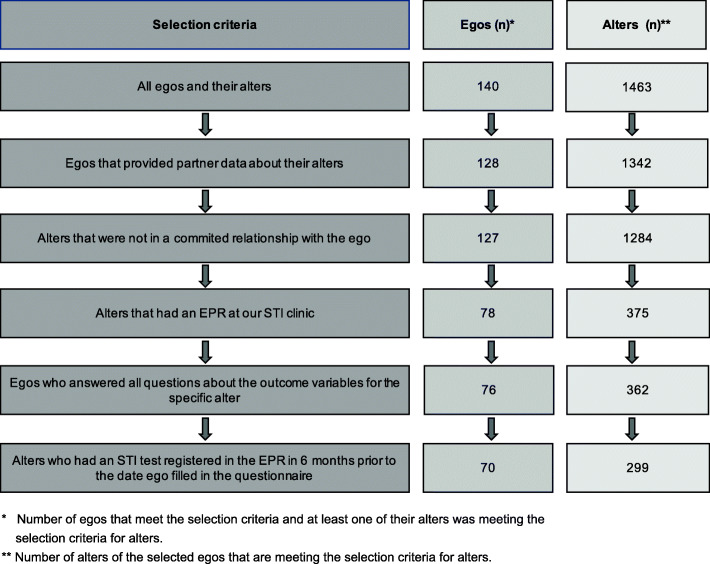
Flowchart depicting the process of selecting egos and alters

The questions that were used to assess the outcome variables of this study were posed to only a subset of the sample of the cohort study, the first two waves of the cohort study in 2009 and 2010. Therefore, only the 140 egos who had responded to these questions were included in the sample. The sample was further restricted to egos and alters who met the inclusion criteria described in Fig. 1. Egos (*n* = 70) with at least one alter who met the inclusion criteria for alters were included. The primary reason for the exclusion of egos was the absence of their alters’ electronic patient records (EPRs) in our STI clinic (see Fig. 1).

We compared the subsets of included and excluded egos and alters on their sociodemographic characteristics, swing behaviours, and outcome variables to assess the generalisability of the findings. The results are presented in Additional file 1.

### Data

We used two datasets in this study: self-administered questionnaire data and patient data retrieved from the EPR in our clinic. With regard to the first dataset, egos provided individual partner data (i.e. network data). We collected the egocentric network data using a name generator, i.e. a list of all their swing sex partners, in the past six months. For every alter in the name generator the egos completed a partner data questionnaire that assessed each of his or her altars’ characteristics, sexual behaviours, number of sex partners, type of swing relationship, and STI status during the past six months. They also completed a self-administered questionnaire that assessed their personal characteristics and sexual behaviours including their educational level, the number of years for which they had been swingers (i.e. swing duration), the frequency with which they engaged in swinging, swinging venues, their number of sex partners, sexual preferences, and drug or alcohol use during swinging.

The second dataset (i.e. patient data from the EPR), which was collected during STI consultations in our clinic, included information about alters’ self-reported characteristics, their behaviours, and STI test results. The actual STI diagnostic data of the egos were also extracted from their EPR.

To address the aim of this study, we compared the network and EPR data. The network and EPR data served as operationalisations of the ego-estimated and self-reported behaviours of alters, respectively.

### Outcome variables

The outcome variables that were examined to assess the level of agreement between the network and EPR data were bisexual behaviour, number of sex partners, and STI status. These variables served as proxies for STI risk.

Heterosexual behaviour was defined as the act of having sex with only partners of the opposite sex. Bisexual behaviour was defined as the act of having sex with both opposite- and same-sex partners. Bisexual behaviour was considered to have been underestimated if heterosexual behaviour was reported in the network data and bisexual behaviour was reported in the EPR data.

With regard to both the network and EPR data, bisexual behaviour was measured by asking the participant whether the alter had had sex with only men, only women, or both men and women. A nurse registered participant responses to this question in the EPR after posing questions that assessed the different types of sex (including oral sex) that they had engaged in with both men and women. Depending on the gender of the alter, the response was coded as ‘heterosexual behaviour’ if the alter had had sex with only members of the opposite gender and as ‘bisexual behaviour’ if the alter had had sex with both women and men.

The number of sex partners was defined as the number of male and female sex partners that one had had during the past six months. A higher number of sex partners was considered to be indicative of higher STI risk. The number of sex partners was considered to have been underestimated if the network data yielded a lower figure than the EPR data did.

The following questionnaire item (i.e. network data) was used to measure egos’ estimations of the number of sex partners that their alters had: ‘Provide an estimation of the number of sex partners that you think [alter name] has had during the past 6 months’. They provided separate responses for their male and female alters. With regard to the EPR data, the following question was posed: ‘How many sex partners have you had during the past 6 months?’. There was no provision to record separate responses for their male and female sex partners.

Accuracy was considered to be less important for higher numbers of sex partners. In other words, STI risk would be higher among those with five sex partners than among those with only one sex partner. However, the difference in STI risk between those with 70 and 60 sex partners is unlikely to be significant. Therefore, we defined the overestimation and underestimation of the number of sex partners based on a range and estimation margin. Higher numbers of sex partners permitted a larger estimation margin. The number of sex partners was classified into the following ranges: < 10, 10–20, 20–50, and > 50. A 20% margin yielded maximum margins of 2, 4, 10, and 20, respectively.

The STI status was defined as positive if at least one STI was diagnosed during the past six months. Using the network data, the STI status of alters was classified as follows: the alter does not have an STI, the alter does have an STI, the ego was not aware of the alter’s STI status. We used STI test results that were retrieved from the EPR data to ascertain alters’ actual STI statuses. STI status was considered to have been underestimated if it was negative in the network data but positive in the EPR data.

The following questionnaire item (i.e. network data) was used to assess alters’ STI status: ‘Did this alter have an STI during the past 6 months?’. The egos could record their responses on a five-point scale that included the following response options: ‘I know for sure that he did not have an STI’, ‘I presume that he did not have an STI’, ‘I don’t know if he had an STI’, ‘I presume that he had an STI’, and ‘I am sure he had an STI’. These five response options were collapsed into the three aforementioned estimations. The first two response options were combined into the estimation that the alter did not have an STI, and the last two response options were combined into the estimation that the alter did have an STI. The remaining response stated that the ego did not know the alter’s STI status.

STI test results across the preceding seven months were extracted from the EPR dataset. They were summed to ascertain the STI status during the past six months. To determine the STI status, we chose to include a one-month window period to account for a delay of two or three weeks between STI testing and the delivery of results. The STI tests included regular screening for *Chlamydia trachomatis*, *Neisseria gonorrhoeae*, *Treponema pallidum* (syphilis), human immunodeficiency virus (HIV), and hepatitis B virus, as well as clinically confirmed diagnoses of *condylomata acuminata* and *herpes genitalis*. A STI status was defined positive if at least one of the aforementioned STIs was diagnosed during the past six months.

With regard to the network data, the response, ‘I don’t know’, to any question that assessed the outcome variables was considered to be a lack of response and classified into a separate category. In the tables presented in this article, continuous variables (e.g. age, swinging duration and frequency, number of sex partners) are dichotomised based on their median.

### Statistical analysis

We compared the network and EPR data to ascertain the level of agreement with regard to the three outcome variables at the ego-alter dyad level. Chi-squared test was used to assess the level of agreement between the ego-estimated and self-reported behaviours of alters. The data were stratified by the gender of the egos and alters because there were significant differences in the bisexual behaviours of male and female alters (see Tables 1 and 2). All statistical analyses were conducted using SPSS version 24 for Mac (IBM Inc., Somers, NY, USA).

**Table 1:** Outcome variables derived from the network data and EPR data stratified by gender

	Estimation given by egos										EPR alter									
	Male egos (***n*** = 135)					Female egos (***n*** = 164)					Male egos					Female egos				
	Male alters (*n* = 38)		Female alters (*n* = 97)			Male alters (*n* = 85)		Female alters (*n* = 79)			Male alters (n = 38)		Female alters (n = 97)			Male alters (n = 85)		Female alters (n = 79)		
	n	% (95%CI)	n	% (95%CI)	p^#^	n	% (95%CI)	n	% (95%CI)	p^#^	n	% (95%CI)	n	% (95%CI)	p^#^	n	% (95%CI)	n	% (95%CI)	p^#^
**Bisexuality**					**					**					**					**
Heterosexual	20	52.6 (36.7–68.5)	5	5.2 (0.8–9.6)	< 0.001	55	64.7 (54.5–74.9)	6	7.6 (1.8–13.4)	< 0.001	11	28.9 (14.5–43.3)	0	0 (0.0–0.0)	< 0.001	31	36.5 (26.3–46.7)	0	0,0 (0.0–0,0)	< 0.001
Bisexual	18	47.4 (31.5–63.3)	92	94.8 (90.3–99.2)		25	29.4 (19.7–39.1)	73	92.4 (86.6–98.2)		27	71.1 (56.7–85.5)	97	100,0 (100.0–100.0)		54	63.5 (53.3–73.7)	79	100,0 (100.0–100.0)	
Don’t know	0	0,0 (0.0–0.0)	0	0,0 (0.0–0.0)		5	5.9 (0.9–10.9)	0	0.0 (0.0–0,0)											
**Sex partners in past 6 months**																				
< 14 partners	13	34.2 (19.1–49.2)	23	23.7 (15.2–32.2)	0.462	35	41.2 (30.7–51.7)	19	24.1 (14.7–33.5)	0.054	26	68.4 (53.6–83.1)	69	71.1 (62.1–80.1)	0.827	63	74.1 (64.8–83.4)	58	73.4 (63.7–83.1)	0.937
≥ 14 partners	9	23.7 (10.2–37.2)	26	26.8 (18.0–35.6)		22	25.9 (16.6–35.2)	30	38.0 (27.3–48.7)		11	28.9 (14.5–43.3)	24	24.7 (16.1–33.3)		18	21.2 (12.5–29.9)	18	22.8 (13.5–32.1)	
Don’t know	16	42.1 (26.4–57.8)	48	49.5 (39.6–59.5)		28	32.9 (22.9–42.9)	30	38.0 (27.3–48.7)		1	2.6 (−2.5–7.7)	4	4.1 (0.2–8.0)		4	4.7 (0.2–9.2)	3	3.8 (−0.4–8.0)	
**STI status**										**										
No	12	31.6 (16.8–46.4)	46	47.4 (37.5–57.3)	0.114	62	72.9 (63.5–82.3)	33	41.8 (27.3–48.7)	< 0.001	27	71.1 (56.7–85.5)	77	79.4 (71.4–87.4)	0.301	69	81.2 (72.9–89.6)	66	83.5 (75.3–91.7)	0.691
Yes	4	10.5 (0.8–20.2)	14	14.4 (7.4–21.4)		5	5.9 (0.9–10.9)	4	5.1 (0.2–10,0)		11	28.9 (14.5–43.3)	20	20.6 (12.5–28.6)		16	18.8 (10.5–27.1)	13	16.5 (8.3–24.7)	
Don’t know	22	57.9 (42.2–73.6)	37	38.1 (28.4–47.8)		18	21.2 (12.5–29.9)	42	53.2 (42.2–64.2)											

^#^ *p* values (χ2-test)

** = *p* < 0.01, indicates significant differences between males and females

**Table 2:** Agreement measures for the outcome variables at the dyadic level, stratified by gender

	Male egos (n = 135)					Female egos (n = 164)					Total participants (***n*** = 299)				
	Male alters (*n* = 38)		Female alters (*n* = 97)			Male alters (*n* = 85)		Female alters (*n* = 79)			Male alters (*n* = 123)		Female alters (*n* = 176)		
	n	% (95%CI)	n	% (95%CI)	p^#^	n	% (95%CI)	n	% (95%CI)	p^#^	n	% (95%CI)	n	% (95%CI)	p^#^
**Bisexuality**					**					**					**
Underestimation (bisexuality estimated as heterosexuality)	11	28.9 (14.5–43.3)	5	5.2 (0.8–9.6)	< 0.001	34	40.0 (29.6–50.4)	6	7.6 (1.8–13.4)	< 0.001	45	36.6 (28.1–45.1)	11	6.3 (2.7–9.9)	< 0.001
Correctly estimated	25	65.8 (50.7–80.9	90	92.8 (87.7–97.9)		40	47.1 (36.5–57.7)	71	89.9 (83.3–96.5)		65	52.8 (44.0–61.6)	161	91.5 (83.4–95.6)	
Overestimation (heterosexuality estimated as bisexuality)	2	5.3 (−1.8–12.4)	2	2.1 (−0,8–5.0)		6	7.1 (1.6–12.6)	2	2.5 (−0.9–5.9)		8	6.5 (2.1–10.9)	4	2.3 (0.8–4.5)	
No estimation	0	0,0 (0.0–0.0)	0	0,0 (0,0-0,0)		5	5.9 (0.9–10.9)	0	0,0 ( 0.0–0.0)		5	4.1 (0.6–7.6)	0	0.0 ( 0.0–0.0)	
**Number of sex partners**															
Underestimation (number of sex partners estimated too low)	7	18.4 (6.1–30.7)	10	10.3 (4.3–16.3)	0.205	11	13.3 (6.1–20.5)	6	7.6 (1.8–13.4)	0.599	18	14.9 (8.6–2.1)	16	9.1 (4.9–13.3)	0.121
Correctly estimated	5	13.2 (2.4–23.9)	5	5.2 (0.8–9.6)		11	13.3 (6.1–20.5)	8	10.3 (3.6–17.0)		16	13.2 (7.2–19.2)	13	7.5 (3.6–11.4)	
Overestimation (number of sex partners estimated too high)	10	26.3 (12.3–40.3)	33	34.4 (24.9–43.9)		33	39.8 (29.4–50.2)	34	43.6 (32.7–54.5)		43	35.5 (27.0–44.0)	67	38.5 (31.3–45.7)	
No estimation	16	42.1 (26.4–57.8)	48	50.0 (40.0–60.0)		28	33.7 (23.7–43.7)	30	38.5 (27.8–49.2)		44	36.4 (27.9–44.9)	78	44.8 (37.5–52.1)	
**STI status**										**					*
Underestimation (STI positive, estimated STI negative)	1	2.6 (−2.5–7.7)	10	10.3 (4.3–16.3)	0.258	7	8.2 (2.4–14.0)	6	7.6 (1.8–13.4)	< 0.001	8	6.5 (2.1–10.9)	16	9.1 (4.9–13.3)	0.046
Correctly estimated STI positive	2	5.3 (−1.8–12.4)	6	6.2 (1.4–11.0)		3	3.5 (−0.4–7.4)	2	2.5 (−0.9–5.9)		5	4.1 (0.6–7.6)	8	4.5 (1.4–7.6)	
Correctly estimated STI negative	11	28.9 (14.5–43.3)	36	37.1 (27.5–46.7)		55	64.7 (54.5–74-9)	27	34.2 (23.7–44.7)		66	53.7 (44.9–62.5)	63	35.8 (28.7–42.9)	
Overestimation (STI negative, estimated STI positive)	2	5.3 (−1.8–12.4)	8	8.2 (2.7–13.7)		2	2.4 (−0.9–5.7)	2	2.5 (− 0.9–5.9)		4	3.3 (0.1–6.5)	10	5.7 (2.3–9.1)	
No estimation	22	57.9 (42.2–73.6)	37	38.1 (28.4–47.8)		18	21.2 (12.5–29.9)	42	53.2 (42.2–64.2)		40	32.5 (24.2–40.8)	79	44.9 (37.6–52.2)	

^#^ *p* values (χ2-test)

* = *p* < 0.05, ** = *p* < 0.01, indicates significant differences between males and females

### Ethics

Medical ethical approval to conduct the SWAP study was granted by the Medical Ethics Committee of the Maastricht University Medical Centre. As required by this committee, written informed consent was obtained from all participants prior to data collection. Medical ethics approval to use the coded EPR data was granted by the same committee. As approved by the committee, all attendees of our clinic gave permission by opt-out consent. A medical professional in the clinic assigned codes to the swingers and linked them to the datasets before anonymising them. We performed the data analysis in a fully anonymised and de-identified manner.

## Results

### Egos

Of the 70 egos, 54 and 46% of them were women and men, respectively. Their median age was 43 years (range = 27–60). Their swinging frequency over the past six months ranged from 1 to 50 (median = 10), and they had had a median of 14 sex partners (range = 1–83) during the past six months. Table A in Additional file 1 summarises the demographic characteristics, swinging behaviours, and STI statuses of the egos. Nine egos (13%) were diagnosed with an STI in the past six months: 7 (10%) with *Chlamydia trachomatis* and 2 (2.9%) with *Neisseria gonorrhoeae*. All egos were tested negative for HIV, *Treponema pallidum* (syphilis) and Hepatitis B. *Condylomata acuminata* and *Herpes genitalis* were not diagnosed.

### Alters

Of the 299 alters, 59 and 41% were women and men, respectively. Their median age was 44 years (range = 21–60). Table B in Additional file 1 summarises their characteristics and swinging behaviours as perceived by the egos. Table 1 presents the three outcome variables (i.e. bisexual behaviour, number of sex partners, and STI status) derived from the network and EPR data. Sixty (20%) of the alters were diagnosed with an STI in the past six months: 23 (7.7%) with *Chlamydia Trachomatis*, 29 (10%) with *Neisseria gonorrhoeae*, 11 (3.7%) with *Condylomata acuminata* and 1 (0.3%) with Herpes genitalis. All alters were tested negative for HIV, *Treponema pallidum* (syphilis) and Hepatitis B. Table 2 presents the level of agreement at the dyadic level.

### Alters’ bisexual behaviour

Both male and female egos were significantly better at estimating the bisexual behaviours of female alters than male alters (*p* < 0.01). Egos underestimated the bisexual behaviours of 37% of their male alters. Male egos underestimated the bisexual behaviours of 29% of their male alters.

### Alters’ number of sex partners

Agreement with regard to the number of sex partners did not differ significantly between male and female alters. Analysis of the network data revealed that the alters had had a median of 13 sex partners (range = 0–230) during the past six months. In contrast, analysis of the EPR data revealed that they had had a median of 8 sex partners (range = 2–270) during the past six months. The median discrepancy in the number of sex partners was 7 (range = 0–215). Overall, when egos did provide an estimation, they overestimated the number of sex partners of 54 and 68% of male and female alters, respectively.

### Alters’ STI status

Agreement with regard to alters’ STI status differed significantly between male and female alters. When an estimation was provided, the STI status of female alters was more frequently underestimated (17%) than that of male alters (9.7%). The negative STI status of male alters (80%) was more frequently correctly estimated than that of female alters (65%). Egos underestimated the STI status of 40% of the 60 alters who had been diagnosed with an STI during the past six months, correctly estimated the STI status of 22% of these alters, and provided no estimation for 38% of these alters. Overall, when they did provide an estimation, they underestimated the positive STI status of 62% of their male alters and 67% of their female alters.

## Discussion

In this study, we adopted a network approach to examine how accurately swingers could estimate the sexual behaviour, number of sex partners, and STI status (i.e. risk factors for STIs) of their swing sex partners. The main findings of this study are that egos underestimated the bisexual behaviour of male alters, overestimated the number of alters’ sex partners, and underestimated their positive STI status.

Although a majority of egos correctly estimated the bisexual behaviour of their alters, a sizeable proportion of them underestimated the bisexual behaviour of male alters. When compared to past findings, lower percentages of the underestimation of the bisexual behaviour of male alters emerged in this study. Ellen et al. [7] found that 86% of women underestimated their male sex partner having sex with men. In comparison, only 47% of the female egos in this study underestimated the bisexual behaviour of their male alters. Surprisingly, male egos estimated that almost one-third of their male alters had sex only with women. We expected egos to estimate that 100% of their same-sex alters would engage in bisexual behaviour because these alters were their sex partners. A possible explanation for this discrepancy is that swingers often do not regard oral sex as sex.

In this study, rates of agreement regarding the number of sex partners that alters had was low. However, in contradistinction to past findings, [2] egos overestimated the number of sex partners that their alters had: Specifically, they overestimated the number of partners of 54 and 68% of their male and female alters, respectively. This overestimation of the number of sex partners may be attributable to underreporting in the EPR. Collecting data about sexual behaviours (e.g. our EPR data) is vulnerable to biases and can result in the underreporting of the number of sex partners that one has [5, 6]. Thus, if too low numbers of sex partners were recorded in the EPR data, egos’ estimations may have been accurate estimations instead of overestimations.

A majority of the egos estimated their alters’ STI statuses correctly (as most alters tested negative) but underestimated their positive STI status. They correctly estimated the STI statuses of only 22% of STI-positive alters. Our findings mirror past observations [12]. However, in this study, swingers were slightly more accurate at estimating the STI statuses of their STI-positive alters.

Past studies [2, 3, 7–13, 15] that have examined the level of agreement between sex partners’ sexual behaviours have reported low rates of agreement for behaviours that a couple does not engage in together. Only a few studies [2] have examined the same risk behaviours, as we did among swingers. In contradistinction to these findings, swingers estimated alters’ bisexual behaviours and number of sex partners rather accurately. A possible explanation is that swingers generally swing at clubs and house parties, [21] where they can openly observe the sexual behaviours of their swing sex partners. However, the stigma surrounding STIs and reluctance to notify one’s partners can contribute to the underestimation of a positive STI status.

The conclusion that swingers underestimate the positive STI status of their alters has implications for STI prevention. In a past study [28] that was conducted within the same population of swingers, STI testing and partner notification emerged as the norm among a majority of the participants. Therefore, relatively accurate estimations of alters’ positive STI statuses were expected. Most egos should have been notified by their alters, as half of them had gone on more than one date with their alters and had shared a swing relationship that exceeded the period of partner notification for most STIs (see Table B in Additional file 1). Thus, swingers’ intentions to notify their partners about an STI diagnosis [28] does not seem to necessarily lead to an actual notification. This suggests that infected swingers find it difficult to notify their sex partners about an STI diagnosis.

Swingers’ tendency to underestimate their alters’ positive STI statuses can cause them to underestimate their own STI risk. Several studies [2, 8, 12, 29, 30] have reported a similar tendency to underestimate STI risk and found that it negatively influences STI testing behaviours and the adoption of preventive measures [2, 29]. Therefore, our findings have important implications for STI prevention. Further attention should be paid to proper partner notification and the promotion of STI testing among swingers.

Our study is unique because it focused on a hidden population: swingers. Further, we compared the behaviours of casual swing sex partners rather than couples in committed relationships. These swing sex partnerships characterise the swinger population. However, our study also has several limitations. First, the findings may not be generalisable to all swingers because the study used a convenience sample of swingers who visited our clinic [5]. Evidently, swingers who visit an STI clinic to be tested will be more aware of their STI risk than the general swinger population. Moreover, we included only a third of the alters and half of the egos in our sample (see Fig. 1). It is possible that some differences in the outcomes were present but not statistically significant in our study due to the current sample size of our convenience sample. The main reason for the exclusion of egos and alters was that many alters were not or could not be identified as clients of our clinic. A comparison of the included and excluded alters (see Additional file 1) revealed that the egos and included alters had gone on more dates together and had been in swing relationships for longer durations. These findings lead one to expect that egos would be more aware of the STI risk of their included alters. However, logistic regression analysis (data not presented) revealed that there was no significant relation between these variables and the outcome variables.

Second, the outcome variables were estimated within a six-month temporal framework. Thus, as noted earlier, recall biases may adversely influence the information contained in both datasets. This limitation may be attributable to the fact that many egos did not provide an estimation of alters’ number of sex partners and STI status. Furthermore, if an alter had contracted an STI and received treatment prior to his/her first swing date with an ego, then the underestimation of a positive STI status may have actually been correct. Because a majority of the egos and alters had gone on more than one date and had been in a swing relationship for more than one year, these underestimations are unlikely to be erroneous.

Finally, the data used in this study were collected between 2009 and 2010. Nevertheless, there is no reason to expect that swingers’ levels of awareness about their alters’ behaviours would have significantly changed since the time of data collection. There is no STI prevention program for swingers in the Netherlands. Therefore, the findings of this study are unlikely to have been adversely influenced by the aforementioned limitation.

## Conclusion

Swingers underestimated the bisexual behaviours of their male swing sex partners, overestimated the number of alters’ sex partners, and underestimated their positive STI status. By underestimating the positive STI statuses of their partners, swingers may underestimate their own STI risk and fail to implement preventive measures. The latter finding has important implications for STI prevention. Tailored interventions for swingers that include the promotion of STI testing and actual partner notification are needed for this hidden sexual subpopulation.

## Supplementary Information

**Additional file 1.** A comparison of the excluded and included egos and alters alters in terms of sociodemographic characteristics, swing behaviours, and outcome variables to assess the generalisability of the findings.

**Additional file 2.** Translation of the for the current study relevant questions from the (originally Dutch) questionnaires.

## Data Availability

The datasets used and analysed during the current study are available from the corresponding author on reasonable request.

## Abbreviations

EPR: Electronic patient record
HIV: Human immunodeficiency virus
STI: Sexually transmitted infection
