# Supplementary material for: How aware are swingers about their swing sex partners’ risk behaviours, and sexually transmitted infection status?
Source: BMC Infect Dis. 2021 Feb 12;21:172. doi: 10.1186/s12879-021-05813-5 (PMC7881639; doi:10.1186/s12879-021-05813-5)
Supplement: Supplementary file 1 — Additional file 1. A comparison of the excluded and included egos and alters alters in terms of sociodemographic characteristics, swing behaviours, and outcome variables to assess the generalisability of the findings. [file 12879_2021_5813_MOESM1_ESM.docx]

**SUPPLEMENTARY APPENDIX**

**Appendix 1. A comparison of included and excluded egos and alters.**

We compared excluded and included egos and alters alters in terms of sociodemographic characteristics, swing behaviours, and outcome variables to assess the generalisability of the findings.

Table A presents the demographic characteristics and swing behaviours of the included and excluded egos, which were assessed using questionnaires. In addition, their sexually transmitted infection (STI) status, which was ascertained based on the electronic patient record (EPR) data, is also presented in this table. When compared to the excluded egos, the included egos engaged in swinging more frequently, had more sex partners, and used drugs more frequently.

The included and excluded alters were compared on demographic characteristics, sexual preferences, number of sex partners, STI status, and the swing relationship, as estimated by the egos (n = 70). The results are presented in Table B. When compared to the excluded alters, the included alters had met their egos at house parties more frequently, been in swing relationships for longer durations of time, gone on more dates with their egos, and shared drugs more frequently (Table A).

The 70 egos who participated in this study had 896 alters, 299 (33%) of whom were included because they had an EPR in our clinic and met the inclusion criteria (see Fig 1). The median number of included alters per ego was 6 (range=1–27). The egos estimated the behaviours of the included and excluded alters differently (Table B). Specifically, they more frequently reported that the excluded alters engaged in only heterosexual behaviours and had fewer sex partners and less frequently reported that they had STIs.

**Table A. Comparisons of the sociodemographic characteristics, swing behaviours, and STI status of included and excluded egos.**

| **Participants** | **Included**  **egos**  **(n=70)** | | | | | | **Excluded**  **egos**  **(n=70)** | | **All**  **egos**  **(n=140)** |  |
| --- | --- | --- | --- | --- | --- | --- | --- | --- | --- | --- |
|  | **Male (n=32)** | | **Female (n=38)** | | **All**  **(n=70)** | |  | |  |  |
|  | % (n) | | % (n) | | % (n) | | % (n) | | % (n) | p^#^ |
| **Demographics** |  | |  | |  | |  | |  |  |
| Gender |  | |  | |  | |  | |  |  |
| Male | 100 (32) | |  | | 46 (32) | | 53 (37) | | 49 (69) | 0.398 |
| Female |  | | 100 (38) | | 54 (38) | | 47 (33) | | 51 (71) |  |
| Age |  | |  | |  | |  | |  |  |
| ≤43 years | 47 (15) | | 63 (24) | | 56 (39) | | 53 (37) | | 54 (76) | 0.734 |
| >43 years | 53 (17) | | 37 (14) | | 44 (31) | | 47 (33) | | 46 (64) |  |
| Education |  | |  | |  | |  | |  |  |
| Low | 22 (7) | | 21 (8) | | 21 (15) | | 16 (11) | | 19 (26) | 0.385 |
| Middle/high | 78 (25) | | 79 (30) | | 79 (55) | | 84 (59) | | 81 (114) |  |
| Regular relationship |  | |  | |  | |  | |  |  |
| No | 3.1 (1) | | 2.6 (1) | | 2.9 (2) | | 10 (7) | | 6.4 (9) | 0.085 |
| Yes | 97 (31) | | 97 (37) | | 97 (68) | | 90 (63) | | 94 (131) |  |
| **Swing behaviour** |  | |  | |  | |  | |  |  |
| Swing duration |  | |  | |  | |  | |  |  |
| < 3 years | 41 (13) | | 40 (15) | | 40 (28) | | 34 (24) | | 37 (52) | 0.484 |
| ≥ 3 years | 59 (19) | | 61 (23) | | 60 (42) | | 66 (46) | | 63 (88) |  |
| Venues past 6 months |  | |  |  |  | |  |  |  |  |
| House parties only | 56 (18) | | 47 (18) | | 51 (36) | | 41 (29) | | 46 (65) | 0.236 |
| Other locations | 44 (14) | | 53 (20) | | 49 (34) | | 59 (41) | | 54 (75) |  |
| Swing frequency in past 6 months |  | |  | |  | |  | |  | ** |
| < 10 times | 47 (15) | | 50 (19) | | 49 (34) | | 70 (49) | | 59 (83) | 0.010 |
| ≥ 10 times | 53 (17) | | 50 (19) | | 51 (36) | | 30 (21) | | 41 (57) |  |
| Sex partners past 6 months |  | |  | |  | |  | |  | ** |
| < 14 sex partners | 53 (17) | | 45 (17) | | 49 (34) | | 77 (54) | | 63 (88) | <0.001 |
| ≥ 14 sex partners | 47 (15) | | 55 (21) | | 51 (36) | | 23 (16) | | 37 (52) |  |
| Sex with men/women |  |  |  |  |  |  |  | |  |  |
| Heterosexual | 66 (21) | | 7.9 (3) | | 34 (24) | | 51 (36) | | 43 (60) | 0.040 |
| Bisexual | 34 (11) | | 92 (35) | | 66 (46) | | 49 (34) | | 57 (80) |  |
| Drugs during swinging |  | |  | |  | |  | |  | ** |
| No | 19 (6) | | 29 (11) | | 24 (17) | | 57 (40) | | 41 (57) | <0.001 |
| Yes | 81 (26) | | 71 (27) | | 76 (53) | | 43 (30) | | 59 (83) |  |
| Alcohol during swinging |  | |  | |  | |  | |  |  |
| No | 53 (17) | | 53 (20) | | 53 (37) | | 44 (31) | | 49 (68) | 0.310 |
| Yes | 47 (15) | | 47 (18) | | 47 (33) | | 56 (39) | | 51 (72) |  |
| **STI** |  | |  | |  | |  | |  |  |
| STI in past 6 months (EPR data)ˆ |  | |  | |  | |  | |  |  |
| No | 94 (30) | | 82 (31) | | 87 (61) | | 87 (60) | | 87 (121) | 0.974 |
| Yes | 6.3 (2) | | 18 (7) | | 13 (9) | | 13 (9) | | 13 (18) |  |
| Chlamydia trachomatis |  | |  | |  | |  | |  |  |
| No | 97 (31) | | 84 (32) | | 90 (63) | | 93 (64) | | 91 (127) | 0.563 |
| Yes | 3.1 (1) | | 16 (6) | | 10 (7) | | 7.2 (5) | | 8.6 (12) |  |
| Neisseria gonorrhoeae |  | |  | |  | |  | |  |  |
| No | 97 (31) | | 97 (37) | | 97 (68) | | 96 (66) | | 96 (134) | 0.637 |
| Yes | 3.1 (1) | | 2.6 (1) | | 2.9 (2) | | 4.3 (3) | | 3.6 (5) |  |
| Condylomata acuminata |  | |  | |  | |  | |  |  |
| No | 0 (0) | | 0 (0) | | 0 (0) | | 99 (68) | | 99 (138) | 0.312 |
| Yes | 0 (0) | | 0 (0) | | 0 (0) | | 1.4 (1) | | 0.7 (1) |  |

^#^ p values (χ2-test) of the comparison of included and excluded participants

*= p < 0.05, **= p < 0.01, indicates significant differences between included and excluded participants

ˆ No HIV, Treponema pallidum (syphilis), hepatitis B and herpes genitalis were diagnosed in the past 6 months

**Table B. Comparisons between included and excluded alters on sociodemographic characteristics, sexual preferences, number of sex partners, STI status, and the nature of the swing relationship, as estimated by egos (n = 70).**

| **Alters** | **Included**  **alters**  **(n=299)** | **Excluded**  **alters**  **(n=597)** | **All**  **alters**  **(n=896)** |  |
| --- | --- | --- | --- | --- |
|  | % (n) | % (n) | % (n) | p^#^ |
|  |  |  |  |  |
| Gender |  |  |  | ** |
| Male | 41 (123) | 46 (274) | 44 (397) | 0.013 |
| Female | 59 (176) | 52 (311) | 54 (487) |  |
| Missing | 0 (0) | 2.0 (12) | 1.3 (12) |  |
| Age |  |  |  | * |
| ≤43 years | 49 (147) | 50 (299) | 50 (446) | 0.002 |
| >43 years | 51 (151) | 45 (271) | 47 (422) |  |
| Don’t know | 0.3 (1) | 4.5 (27) | 3.1 (28) |  |
| Sex with men/women |  |  |  | ** |
| Heterosexual | 29 (86) | 38 (227) | 35 (313) | <0.001 |
| Bisexual | 70 (208) | 54 (323) | 59 (531) |  |
| Don’t know | 1.7 (5) | 7.9 (47) | 5.8 (52) |  |
| Sex partners past 6 months |  |  |  | ** |
| < 14 sex partners | 30 (90) | 43 (258) | 39 (348) | <0.001 |
| ≥ 14 sex partners | 29 (87) | 24 (144) | 26 (231) |  |
| Don’t know | 41 (122) | 33 (195) | 35 (317) |  |
| STI past 6 months |  |  |  | ** |
| No | 51 (153) | 50 (297) | 50 (450) | <0.001 |
| Yes | 9.0 (27) | 1.8 (11) | 4.2 (38) |  |
| Don’t know | 40 (119) | 48 (289) | 46 (408) |  |
| Type of swing partner |  |  |  | ** |
| Regular | 49 (146) | 30 (177) | 36 (323) | <0.001 |
| Loose | 45 (133) | 63 (377) | 57 (510) |  |
| Missing | 6.7 (20) | 7.2 (43) | 7.0 (63) |  |
| Duration of swing relationship |  |  |  | ** |
| ≤ 12 months | 62 (184) | 72 (428) | 68 (612) | <0.001 |
| >12 months | 33 (99) | 18 (110) | 23 (209) |  |
| Missing | 5.4 (16) | 9.9 (59) | 8.4 (75) |  |
| Venue house party |  |  |  | ** |
| No | 9.7 (29) | 27 (159) | 21 (188) | <0.001 |
| Yes | 90 (270) | 73 (436) | 79 (706) |  |
| Missing | 0 (0) | 0.3 (2) | 0.2 (2) |  |
| Number of dates with alter |  |  |  | ** |
| 1 date | 37 (111) | 54 (320) | 48 (431) | <0.001 |
| ≥ 2 dates | 63 (188) | 42 (249) | 49 (437) |  |
| Missing | 0 (0) | 4.7 (28) | 3.1 (28) |  |
| Drugs during swinging with alter |  |  |  | ** |
| No | 9.4 (28) | 32 (191) | 24 (219) | <0.001 |
| Yes | 77 (230) | 53 (318) | 61 (548) |  |
| Missing | 14 (41) | 15 (88) | 14 (129) |  |

^#^ p values (χ2-test)

*= p < 0.05, **= p < 0.01, indicates significant differences between included and excluded alters
