# Supplementary material for: How aware are swingers about their swing sex partners’ risk behaviours, and sexually transmitted infection status?
Source: BMC Infect Dis. 2021 Feb 12;21:172. doi: 10.1186/s12879-021-05813-5 (PMC7881639; doi:10.1186/s12879-021-05813-5)
Supplement: Supplementary file 2 — Additional file 2. Translation of the for the current study relevant questions from the (originally Dutch) questionnaires. [file 12879_2021_5813_MOESM2_ESM.docx]

**Additional file 2**

**Questionnaires**

The for the current study relevant questions in the (originally Dutch) questionnaires are here translated.

We used data from three different questionnaires for the current study:

1. Ego questionnaire: a questionnaire about ego’s own characteristics and behaviour.
2. Name generators: lists of all their swing sex partners (alters) in the past 6 months.
3. Alter questionnaire: a questionnaire per alter about alter’s characteristics and behaviour.

**EGO questionnaire:**

|  | **Dutch questionnaire** | | **English translation** | |
| --- | --- | --- | --- | --- |
| **Nr.** | **Question** | **Answer options** | **Question** | **Answer options** |
| 1 | Wat is uw geslacht? | - Man - Vrouw | What is your sex? | - Male - Female |
| 2 | Wat is uw geboortedatum? | dd-mm-yyyy | What is your date of birth? | dd-mm-yyyy |
| 3 | Wat is de hoogste opleiding die u heeft afgemaakt of nog mee bezig bent? | - Geen opleiding - Lager onderwijs - Voorbereidend beroepsonderwijs - Middelbaar beroepsonderwijs - Hoger algemeen en voorbereidend onderwijs - Hoger beroepsonderwijs - Wetenschappelijk onderwijs - Anders, namelijk … | What is the highest level of education you have finished or are currently doing? | - No education - Elementary School - Different types and levels of Dutch education comparable with (junior and senior) High School - University - Something else, namely… |
| 4 | Wat is op u van toepassing? | - Ik ben vrijgezel - Ik ben getrouwd/heb een vaste partner en woon niet samen - Ik ben getrouwd/heb een vaste partner en woon wel samen - Ik ben weduwe/weduwnaar en heb geen nieuwe partner - Anders, namelijk … | What applies to you? | - I am single - I am married/have a steady partner and we do not live together - I am married/ have a steady partner and we live together - I am widow/ widower and do not have a new partner. - Something else, namely … |
| 5 | Hoe lang swingt u? | … jaar en … maanden | Since when do you swing? | ….. years and …. months |
| 6 | Heeft u de afgelopen 6 maanden swingerswebsites op internet bezocht? | - Nee - Ja | Did you visit swingers’ websites during the past 6 months? | - No - Yes |
| 7 | Heeft u een thuisdate gehad de afgelopen 6 maanden? | - Nee - Ja, bij mij thuis - Ja, bij anderen thuis - Ja, zowel bij mij als bij anderen thuis | Did you had a (swingers) home date/party during the past 6 months? | - No - Yes, at my place - Yes, at someone else’s place - Yes, both at my place and someone else’s place |
| 8 | Heeft u de afgelopen 6 maanden (een) parenclub(s) bezocht? | - Nee - Ja | Did you visit a swingers club during the past 6 months? | - No - Yes |
| 9 | Heeft u professioneel georganiseerde erotische feesten of swingfeesten bezocht de afgelopen 6 maanden? | - Nee - Ja | Did you attend a professionally organized erotic party or swingers’ party during the past 6 months? | - No - Yes |
| 10 | Hoe vaak heeft u geswingd de afgelopen 6 maanden? | In total … keer | How often did you swing during the past 6 months? | In total ….. times. |
| 11 | Met personen van welk geslacht heeft u seks gehad de afgelopen 6 maanden | - Alleen met mannen - Alleen met vrouwen - Met zowel mannen als vrouwen | Whom (persons of what sex) did you have sex with during the past 6 months? | - Only with men - Only with women - With both men and women. |
| 12 | Met hoeveel verschillende personen had u de afgelopen 6 maanden seks tijdens het swingen. | … mannen  … vrouwen | With how many persons did you had sex during swinging in the past 6 months. | … men  … women |
| 13 | Gebruikte u de afgelopen 6 maanden drugs of andere middelen? (meerdere opties mogelijk) | - Nee - Ja, tijdens het swingen - Ja, tijdens seks met mijn vaste partner - Ja, tijdens seks met andere sekscontacten - Ja, maar niet voor of tijdens seks | Did you use drug or other substances during the past 6 months? (more answers possible) | - No - Yes, during swinging - Yes, during sex with my steady partner - Yes, during sex with other sex contacts - Yes, but not before or during sex |
| 14 | Dronk u de afgelopen 6 maanden alcohol? | - Nee - Ja, tijdens het swingen - Ja, tijdens seks met mijn vaste partner - Ja, tijdens seks met andere sekscontacten - Ja, maar niet voor of tijdens seks | Did you use alcohol during the past 6 months? | - No - Yes, during swinging - Yes, during sex with my steady partner - Yes, during sex with other sex contacts - Yes, but not before or during sex |

**Name generators**

(translated example, but the name generator used had unlimited options to name couples and singles).

Name generator couples:

With how many couples did you swing during the past 6 months? With … couples (give a number).

Give for every person in a steady relationship (couple) you did swing with in the past 6 months the information in the table below.

| Couple nr | Person | Sex (M or F) | First name / swingers nick name | Place of residence | Did you had sex with this person during the past month (y/n) | Did you had sex with this person during the past 6 months (y/n) |
| --- | --- | --- | --- | --- | --- | --- |
| Couple 1 | a |  |  |  |  |  |
|  | b |  |  |  |  |  |
| Couple 2 | a |  |  |  |  |  |
|  | b |  |  |  |  |  |
| Couple 3 | a |  |  |  |  |  |
|  | b |  |  |  |  |  |

Name generator steady partner(s) and single persons:

With how many singles did you swing during the past 6 months? With … singles (give a number).

Give for your steady/romantic partner(s) and every single person you did swing with in the past 6 months the information in the table below. Give the information on the steady partner(s) first.

| Person nr | Steady/romantic partner. If yes put X | Sex (M or F) | First name / swingers nick name | Place of residence | Did you had sex with this person during the past month (y/n) | Did you had sex with this person during the past 6 months (y/n) |
| --- | --- | --- | --- | --- | --- | --- |
| Person 1 |  |  |  |  |  |  |
| Person 2 |  |  |  |  |  |  |
| Person 3 |  |  |  |  |  |  |

**Alter questionnaire**

|  | **Dutch questionnaire** | | **English translation** | |
| --- | --- | --- | --- | --- |
| **Nr.** | **Question** | **Answer options** | **Question** | **Answer options** |
| 1 | Wat is het geslacht van deze persoon? | - Man - Vrouw | What is the sex of this person? | - Male - Female |
| 2 | Wat is de (geschatte) leeftijd van deze persoon | … jaar | What is the (estimated) age of this person | … years |
| 3 | Deze persoon is voor mij: | - Een vast swingcontact - Een los swingcontact - Een pay-date - Een eenmalig swingcontact - Mijn vaste partner - Anders, namelijk … | This person is to me: | - A regular swing partner - A loose swing partner - A pay-date - A one-time swing partner - My steady (romantic) partner - Someone else, namely … |
| 4 | Sinds wanneer swingt u samen met deze persoon? | … maand … jaar | Since when do you swing with this person? | … month … year |
| 5 | Hoe vaak heeft u met deze persoon geswingd in de afgelopen 6 maanden? | ... | How often have you been swinging with this person in de past 6 months? | … |
| 6 | Heeft u in de afgelopen 6 maand seks gehad met deze persoon? | - Nee - Ja, alleen tijdens swingdates - Ja, zowel tijdes swingdates als daarbuiten - Ja, maar niet tijdens swingdates | Did you had sex with this person in the past 6 months? | - No - Yes, only during swingdates - Yes, both during swingdates and outside swingdates - Yes, but not during swingdates |
| 7 | Hoe vaak had u seks met deze persoon in de afgelopen 6 maanden? | … | How often did you had sex with this person during the past 6 months? | … |
| 8 | Waar had u in de afgelopen 6 maanden swingdates met deze person? | - Thuis (bij mij of anderen) - Swing/parenclub - Hotel / vakantiehuisje - Swingfeest - Anders, namelijk… | Where did you swing with this person during the past 6 months? | - Home party (at my place or at someone else’s place) - Swingers club - Hotel / holiday home - Swing party - Somewhere else, namely … |
| 9 | Werden tijdens of voor de seks met deze person drug/middelen gebruikt? | - Nee - Ja, door mij - Ja, door deze persoon - Ja, door ons beiden | Did you or this person uses drug/substances before or during sex? | - No - Yes, by me - Yes, by this person - Yes, by both of us |
| 10 | Ik denk dat deze person seks heeft met: | - Alleen mannen - Alleen vrouwen - Zowel mannen als vrouwen | I think/estimate that this person has sex with: | - Only with men - Only with women - With both men and women. |
| 11 | Geen een schatting: hoeveel sekspartners heeft deze persoon gehad de afgelopen 6 maanden | ... mannen  … vrouwen | Give an estimation: how many sex partners did this person had during the past 6 months? | … men  … women |
| 12 | Heeft deze persoon de afgelopen 6 maanden een SOA gehad? | - Ik weet zeker dat hij/zij GEEN SOA heeft gehad - Ik vermoed dat hij/zij GEEN SOA heeft gehad - Ik weet niet of hij/zij een SOA heeft gehad - Ik vermoed dat hij/zij WEL een SOA heeft gehad - Ik weet zeker dat hij/zij WEL een SOA heeft gehad | Did this person had an STI during the past 6 months? | - I know for sure that he/she did not have an STI - I presume that he/she did not have an STI - I don’t know if he/she had an STI - I presume that he/she had an ST - I am sure he/she had an STI'. |
